# Parental acceptance of silver Diamine fluoride application on primary dentition: a systematic review and meta-analysis

**DOI:** 10.1186/s12903-020-01195-3

**Published:** 2020-08-20

**Authors:** Heba Sabbagh, Mashael Othman, Layla Khogeer, Haifa Al-harbi, Amjad Al harthi, Asmaa Abdulgader Yaseen Abdulgader

**Affiliations:** 1 Department of Pediatric Dentistry, Faculty of dentistry, King Abdulaziz University, Jeddah, Saudi Arabia; 2 Saudi Endodontic Board, King Abdulaziz Medical City, National Guard Hospital, Jeddah, Saudi Arabia; 3 Department of Pedodontics, King Abdulaziz University, Jeddah, Saudi Arabia; 4 Faculty of Dentistry, King Abdulaziz University, Jeddah, Saudi Arabia

**Keywords:** Parental acceptance, Silver diamine fluoride, Primary dentition, Dental esthetics, Parental perception

## Abstract

**Background:**

This systematic review of the literature was carried out to assess parental acceptance for silver diamine fluoride (SDF) application and esthetic outcome on their children primary dentition and evaluate factors that might influence their acceptance.

**Methods:**

Our research protocol included a search strategy, inclusion/exclusion criteria, and a data extraction plan. The search engines we used were PubMed, Google Scholar, and Science Direct. Reviewers independently reviewed, determined and carried out quality assessment for included studies using CONSORT (for clinical-trials), and STROBE (for Observational studies). In addition, evidence and recommendation’s strength was conducted using Shekelle et al. system. Subsequently, a meta-analysis was performed to assess the association between parental acceptance for SDF treatment and teeth type, location and child’s cooperation.

**Results:**

Eight studies fulfilled the inclusion criteria. There were statistically significant differences between parental acceptance for SDF usage on posterior teeth compared to anterior teeth (*P* < 0.001, OR: 0.23 and 95% CI: 0.15–0.34) and for SDF usage on anterior teeth of uncooperative compared to cooperative children (*P* < 0.001, OR: 0.27 and 95% CI: 0.17–0.44). Additionally, parent’s acceptance rate for SDF application increased after follow-up visits and education.

**Conclusion:**

Parental acceptance for SDF treatment was significantly related to tooth location, child cooperation and pre-operative instruction.

## Background

Development of dental caries is considered the most prevalent infectious disease worldwide [1]. Internationally, 60–90% of school-going children have dental caries [2]. The consequences of untreated dental caries include pain, absence from school, poor school performance, an increased requirement of general anesthesia during treatment, and an increased treatment cost [3, 4].

A recently developed treatment for dental caries is silver diamine fluoride (SDF) [5, 6]. SDF is a topical medicament that acts as an anticariogenic agent against active carious lesions [7, 8]. It is a noninvasive therapy that does not require any surgical procedures, unlike conventional restorative methods, and is cheaper than other treatment options [9, 10]. Unfortunately, SDF causes black discoloration of carious enamel and dentin, which may be an obstacle to its use [11]. The discoloration affects the aesthetic appearance of a child; thus, many parents may refuse the treatment, causing the dentist to be hesitant in recommending it as a treatment option [12]. Consequently, the parents’ acceptance is the primary barrier in choosing SDF as a treatment for young children [13]. Therefore, the aim of this systematic review of the literature was to assess parental acceptance for SDF treatment on their children primary dentition, and evaluate factors that might influence their acceptance. This review is expected to guide dentists’ in including SDF as a treatment option for pediatric patients. A PICO strategy was constructed to identify the study problem and construct the research question as follow:

1. P: Population: Parents/ caregivers of unaffected healthy children with primary dentition;
2. I: Intervention: SDF for the treatment of carious lesions;
3. C: Comparison: Caregivers of children treated with other restorative materials or different tooth types or tooth location;
4. O: Outcome: Parental acceptance/ perception of SDF application technique or it’s aesthetic outcome.

- Therefore, the review question: “Is the technique for SDF application on primary dentition and it’s aesthetic outcome acceptable by parents?”

## Methods

### Registration

We registered our study in the international prospective register of systematic reviews (PROSPERO 2018) (https://www.crd.york.ac.uk/prospero/), register #CRD42018090776.

### Search strategy

Our systematic review and meta-analysis was conducted according to the Preferred Reporting Items for Systematic Reviews and Meta-Analyses (PRISMA) guideline.

The search was carried out on April 2020, and the keywords used were “silver diamine fluoride” or “silver diammine fluoride” and “parental acceptance” or “parental preference” or “parental satisfaction” or “parental perception”. The search engines were MEDLINE-PubMed, Google Scholar, and Science Direct for reviewing materials (in all languages) from 2000 to April 2020. Five examiners (MAO, LNK, HMA, ASA, AAY) independently evaluated article titles and abstracts for inclusion. If an article was not considered relevant after reading the abstract, then it was not included in the study. The cited references in the included abstracts were screened for any additional published papers on the subject. Finally, full-text articles were screened according to the predefined inclusion and exclusion criteria. Disagreement between authors was resolved through consensus meetings with a sixth author (HS). A request for access was sent to authors who had unpublished data on research related to the parental acceptance of SDF.

### Eligibility criteria

The inclusion criteria were as follows:

1. P: Quantitative studies including clinical trials, case-controlled, cross-sectional, or cohort studies carried out on medically unaffected, healthy children’s primary dentition;
2. I: Studies proposing SDF only for the treatment of carious lesions;
3. C: Parental acceptance of other restorations or comparison of different tooth types and location;
4. O: Parental acceptance of SDF defined as; their perception to its application technique or their aesthetic outcome.

Reviews or qualitative, studies carried out on medically compromised children, studies not related to dentistry or silver diamine fluoride, those that were not articles (eg, book, annual review), those that discussed parental acceptance to their children dental health outcomes other than aesthetics, those that included permanent teeth and those and those not related to parental acceptance were excluded.

The extracted data included study design and setting, sample size, sample distribution per group and eligibility criteria for included participants, and methods used to evaluate the parental acceptance of SDF technique of application and aesthetic “outcomes assessment”.

Only the information specifically related to the research was extracted from the eligible articles. If the same information was reported in more than one article, it was extracted only once.

### Strength of reporting, quality assessment and risk of bias

Strength of reporting, quality assessment and risk of bias were carried out separately and discussed together by the investigators on February 2020. The strengths of reporting of included articles were evaluated using the STROBE (Strengthening the Reporting of Observational Studies in Epidemiology) 2007 checklist for case control, cohort, and cross-sectional studies; and the CONSORT (Consolidated Standards of Reporting Trials) 2010 checklist for clinical trials research. In this systematic review, we evaluated the methodology and result sections (consisting of 22 and 27 items, respectively) of the STROBE and CONSORT checklists. The strengths of reporting were determined by the number of items included or excluded and graded according to the STROBE checklist as 1–7 (poor-strength), 8–15 (moderate-strength), and 16–22 (high-strength) or according to the CONSORT checklist as 1–9 (poor-strength), 10–18 (moderate-strength), and 19–27 (high-strength). If any checklist item was inapplicable for a certain study, a full score was given to that item. The detailed CONSORT and STROBE checklist are shown in Supplemental Tables 1 and 2 (see end of manuscript).

The quality assessment of included studies were assessed using Newcastle-Ottawa Quality Assessment Scale (NOS) [14] for case-control studies; and Oxford Quality Scoring System (Jadad scoring) for clinical trial studies [15]. NOS score ranged from 0 to 9. Studies scored 3 to 6 were graded as “fair,” and less than 3 were graded as “poor” [16]. As for Jadad scoring, the maximum scoring was 5. However, it was not possible in the included studies methodology to blind parents and examiners from the SDF stain. Therefore, we considered scores related to “double blinding” as inapplicable and utilized score three as “high quality” and 2 or less as low quality.

Moreover, the risk of bias for included studies were evaluated according to the Cochrane Handbook for Systematic Reviews of Interventions. It includes five main domains: selection bias, performance bias, attrition bias, reporting bias, and other sources of bias. The following judgments were used: low risk, high risk, or unclear (either for the lack of information or uncertainty over the potential for bias) [17].

In addition, the system developed by Shekelle et al. [18] was used to assess the evidence and strength of recommendation for the included studies. Five review authors (MAO, LNK, HMA, ASA, AYY) independently evaluated the biased assessment risk. Any disagreement was resolved by consensus, and, if necessary, a sixth author (HJS) was consulted.

### Meta-analysis

The meta-analysis was completed on papers that had similar subgrouping for the assessment of factors affecting the parental acceptance of SDF. Parental acceptance was grouped into two categories (acceptable and unacceptable); the parameters labeled “somewhat-acceptable” and “acceptable” were grouped into the “acceptable” group, and the parameters labeled “somewhat-unacceptable” and “unacceptable” were grouped into the “unacceptable” group. The meta-analysis compared the rate of parental acceptance between anterior and posterior teeth and between cooperative and uncooperative children’s anterior and posterior teeth. We used the Review Manager Software (Rev Man 5.1, Cochrane Collaboration) 28 for this analysis and the Mantel-Haenszel method to combine the studies for calculating summary odds ratios and 95% confidence intervals. A statistical test of homogeneity was applied to decide whether the results of separate studies could be combined meaningfully. An inconsistency coefficient (I2 statistic) was computed based on the chi-square test, where a value of more than 50% indicated moderate heterogeneity and a value of more than 75% indicated high heterogeneity. The odds ratios were pooled with a random-effects model. A forest plot displayed ratios with 95% confidence limits for individual studies and a summary estimate of effect. Whenever possible, funnel plot was used to assess publication bias.

## Results

### Search strategy results

**Fig. 1:**
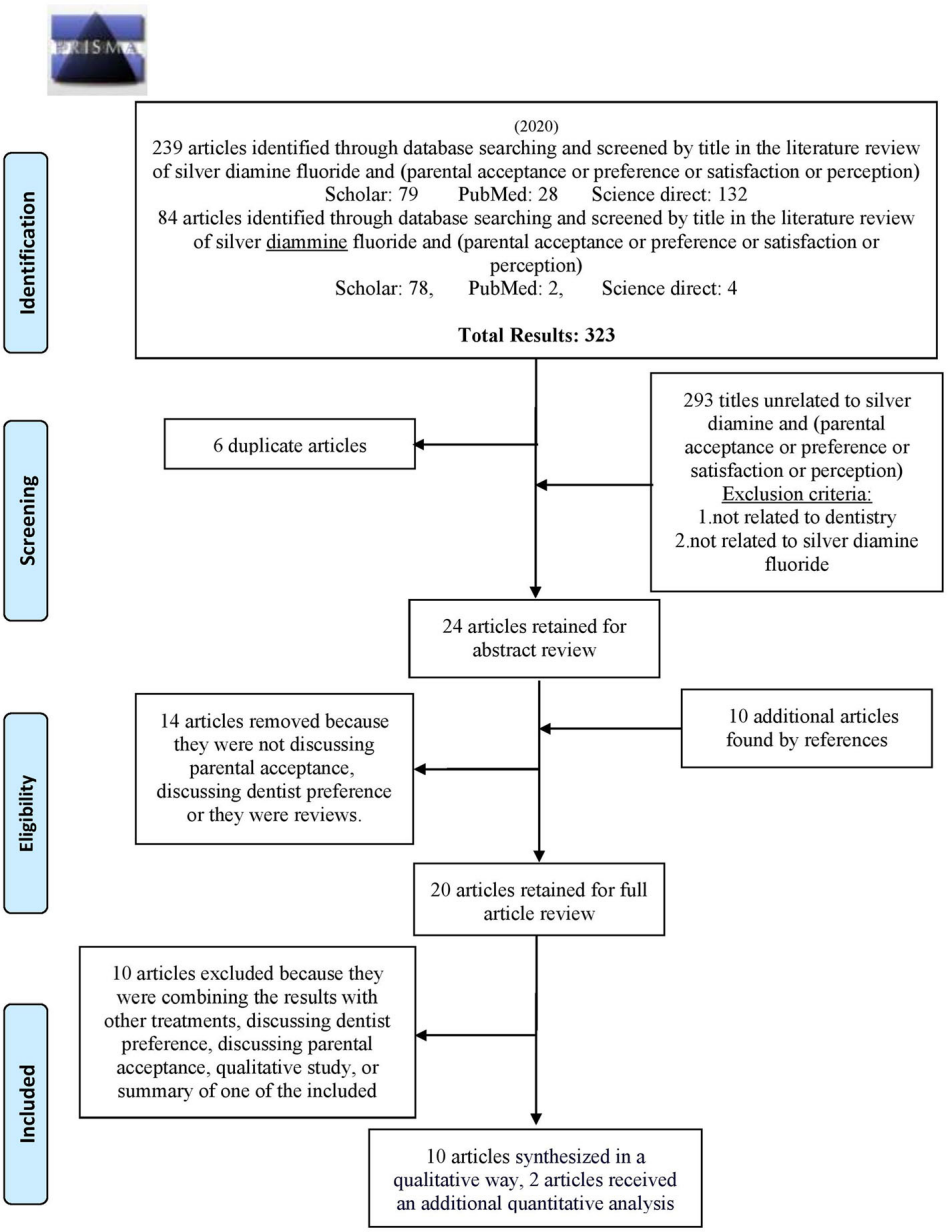
PRISMA 2009 Flow Diagram

Our search strategy yielded 323 hits, including 30 from PubMed, 157 from Google scholar, and 136 from Science Direct. After unrelated titles were excluded and duplicates removed, 24 abstracts remained for reference screening, providing 10 new studies. After all the abstracts were screened according to the inclusion and exclusion criteria; 14 studies were excluded because they either discussed dentists’ acceptance rather than parental acceptance or were reviews. The remaining 20 full articles were screened, and 10 articles were excluded because; (1) they combined results with other treatments (five articles); a summary of one of the included articles (one article); their outcome combined permanent dentition with the primary dentition (3 articles); or was qualitative study (one article). Finally, the study included 10 original articles (Fig. 1).

### Study characteristics

The 10 articles included four cross-sectional investigations [19–22], two clinical trials [12, 23], three randomized control trials (RCTs) [24–26], and 1 case-controlled study [27]. The clinical trial studies considered parental acceptance as a second, non-essential aim [12, 23, 24], thus affecting the quality of the results. Three studies [19–21] compared the parental acceptability rate between the anterior and posterior teeth. All cross-sectional studies utilized photos of primary teeth to assess parental acceptance, while the RCTs measured parental acceptance by the clinical outcome of SDF application. There was only one cross-sectional study that explained SDF to parents without showing them pictures or videos [22]. Questionnaires were used in all the included studies. The characteristics of included studies, their definition of SDF parental acceptance, sample characteristics, design and outcome are listed in Table 1. In addition, parental acceptance definition for each study and the questionnaire scoring system are listed in Table S1.

**Table 1:** Characteristics of included studies

**Reference**	**Site**	**Duration**	**Study design**	**Sample size**	**Children age**	**Methods/*outcome definition*/techniques**	**Groups**	**Results (parental acceptance)**					***P*** **value**
								**Acceptable** **n (%)**	**Somewhat acceptable** **n (%)**	**Not sure/ neutral/ I don’t know** **n (%)**	**Somewhat unacceptable** **n (%)**	**Unacceptable** **n (%)**	
Kumar et al. (2019) [22]	Eight -community health centres affiliated with the NYU Langone Dental Medicine Pediatric Dentistry Residency Program that offers treatment for low SES	May–November 2017	Cross- section	546 caregivers	> 6 y	Questionnaire on parental *perception of the black stain left by the SDF, and their level of comfort* before their children received the SDF treatment (primary teeth)	Dark mark of SDF treatment: Patient < 6 y (*n* = 410)	125 (30.5)	–	–	191 (46.6)	92 (22.4)	No comparison
							Comfort regarding SDF treatment: Patient < 6 y (*n* = 410)	216 (52.7)		125 (30.5)	69 (16.8)		No comparison
Vollú et al. (2019) [26]	Pediatric Dental Clinic of UFRJ, Brazil	June 2016 and August 2017	RCT	67 children 34	2–5 y	Questionnaire on *Parental aesthetic perception* after two weeks of application by questions addressed to caregivers (primary molars)	Test group:(30% SDF) (*n* = 34) Control group: (atraumatic restorative treatment (ART) (*n* = 33).	33 (97.1) 33 (100)			1 (2.9) 0		0.51*
Alshammari et al. (2019) [21]	Saudi Arabia	Not mentioned	Cross- section	222 parents	Not mentioned	Before and after photos with questionnaire on *parental SDF aesthetic acceptance* **(**primary teeth photographs)	Anterior teeth	0 (0)	0 (0)	0 (0)	22 (9.9)	200 (90.1)	*P* < 0.05**
							Posterior teeth	0 (0)	0 (0)	7 (3.2)	63 (28.4)	152 (68.5)	
Duangthip et al. (2018) [25]	37 kindergartens in Hong Kong	Not mentioned	RCT	888 parents	3–4 y	Questionnaire regarding *parental satisfaction with child’s dental appearance* at baseline, 18, 30 months follow-up (primary teeth)	Application of 38% SDF annually: Baseline	3 (1.4)	98 (44.1)	25 (11.3)	**91 (41.0)**	**5 (2.3)**	*P* > 0.05
							18 months follow-up	6 (2.9)	131 (63.3)	29 (14.0)	35 (16.9)	6 (2.9)	
							30 months follow-up after	9 (4.5)	134 (66.3)	24 (11.9)	32 (15.8)	3 (1.59)	
							Application of 12% SDF annually: Baseline	1 (0.5)	79 (35.6)	36 (16.2)	98 (44.1)	8 (3.6) 4 (1.9)	
							18 months follow-up	15 (7.2)	128 (61.8)	22 (10.6)	38 (18.4)		
							30 months follow-up	8 (4.0)	126 (63.6)	21 (10.6)	34 (17.2)	9 (4.5)	
Bagher et al. (2018)	King Abdulaziz University, Jeddah, Saudi Arabi	December 2017–February 2018	Cross-section	104 parents	≤12 y	Before and after photos with questionnaire on *parental preference* (primary teeth)	Anterior primary teeth Posterior primary teeth	17 (16.3) 33 (31.7)	20 (19.2) 37 (35.6)	5 (4.8) 6 (5.8)	19 (18.3) 9 (8.7)	43 (41.3) 19 (18.3)	*P* < 0.05**
							Cooperative Anterior teeth Posterior teeth Uncooperative: Anterior teeth Posterior teeth	10 (12.3) 19 (23.4) 7 (30.4) 14 (60.9)	13 (16) 0 (37) 7 (30.4) 7 (30.4)	2 (2.5) 4 (3.8) 3 (13.0) 2 (8.7)	17 (31) 9 (4.2) 2 (8.7) 0 (0)	39 (48.1) 19 (32.4) 4 (17.4) 0 (0)	
Crystal et al. (2017) [19]	NYU Pediatric Dental Clinic, New York, & private pediatric dentistry clinics, New Jersey, USA	Not mentioned	Cross-section	120 parents	Not mentioned	Before and after treatment sets of photos then questionnaire to evaluate *parents’ acceptance of the aesthetics* (primary teeth photographs)	Anterior teeth Posterior teeth	12*(10.17) 26 (21.67)	23 (19.49) 55 (45.83)	- -	29 (23.73) 13 (10.83)	56 (46.61) 26 (21.67)	*P* < 0.001**
							Cooperative Anterior teeth Posterior teeth Uncooperative: Anterior teeth Posterior teeth	36 (29.7) 81 (67.5) 72 (60.3) 82 (68.5)		- - - -	48 (39.6) 38 (31.5) Not mentioned Not mentioned		
Clements et al. (2017)	Community dental clinic, Oregon, USA	Not mentioned	Clinical study	30 parents	2–5 y	*Parent Acceptability Questionnaire for Silver Diamine Fluoride (SDF) Treatment (discoloration, easy application process, pain, taste*) (primary teeth)	SDF application is an easy process I am comfortable with discoloration of cavities after SDF placement SDF application was pain free for my child The taste of SDF was acceptable to my child	19 (63.3) 16 (53.3) 21 (70.0) 19 (63.3)	8 (26.7) 10 (33.3) 7 (23.3) 7 (23.3)	3 (10.0) 3 (10.0) 2 (6.7) 4 (13.3)	0 1 (3.3) 0 0	0 0 0 0	No comparison
Belotti et al. (2016) [23]	Odontopediatrics clinic in the Federal University of the Espírito Santo, Brazil	Not mentioned	Clinical trial (CT)	14 parents	4–10 y	Photographs were taken before and after SDF treatment. Looking the photographs, parents respond a questionnaire to evaluate the *aesthetics acceptability* (primary molars)	Noticing aesthetic difference Negatively interferes with aesthetics	9* (64.3) 0 (0)		1 (7.1) -	4 (28.6) 14 (100)		No comparison
Zhi et al. (2012) [24]	kindergartens Guangzhou, Guangdong Province in southern China	2007–2009	RCT	212 parents	Not mentioned	Questionnaire on *parent aesthetic satisfaction* at base line and after 24 months (primary teeth)	Gp1: annual application of SDF, Gp2: semi-annual application of SDF Gp3: annual application of glass ionomer	95* (45%) of the parents were satisfied with the appearance of their child’s teeth at the 24-month evaluation					*P* > 0.05
Triches et al. (2009)	UNIPAR’s (State University of Paraná, Brazil) Baby Clinic in the city of Cascavel, PR, Brazil	March–December 2007	Case-control	50 parents	0–3 y	Questionnaire on *parent aesthetic satisfaction* and the effect of instructions about the procedure with post-treatment picture of primary teeth, while the other group showed only a post-treatment picture (primary teeth)	With instructions Without instructions	2 (8) 7 (28)	15 (60) 11 (44)	5 (20) -	1 (4) 6 (24)	2 (8) 1 (4)	0.08*

# Data are reported as no. (%)*only percentage was reported in the study, the number of parents and/or the P value were calculated by the authors, **Significant

*only percentage was reported in the study, the number of parents and/or the *P* value were calculated by the authors

**Significant

### Parental acceptance of SDF and meta-analysis

The included studies reported parental acceptance of SDF according to the location or type of tooth to be restored (anterior vs posterior), patient cooperation (cooperative vs. uncooperative), concentration of SDF, length of follow-up, taste, appearance, application, amount of discomfort, and parental SDF education (Table 1).

### Tooth location (anterior vs. posterior)

**Fig. 2:**
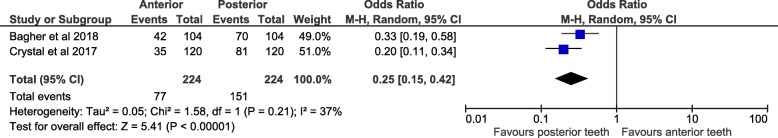
Forest plot for meta-analysis of the association between parental acceptance to silver diamine fluoride and the type of primary teeth

**Fig. 3:**
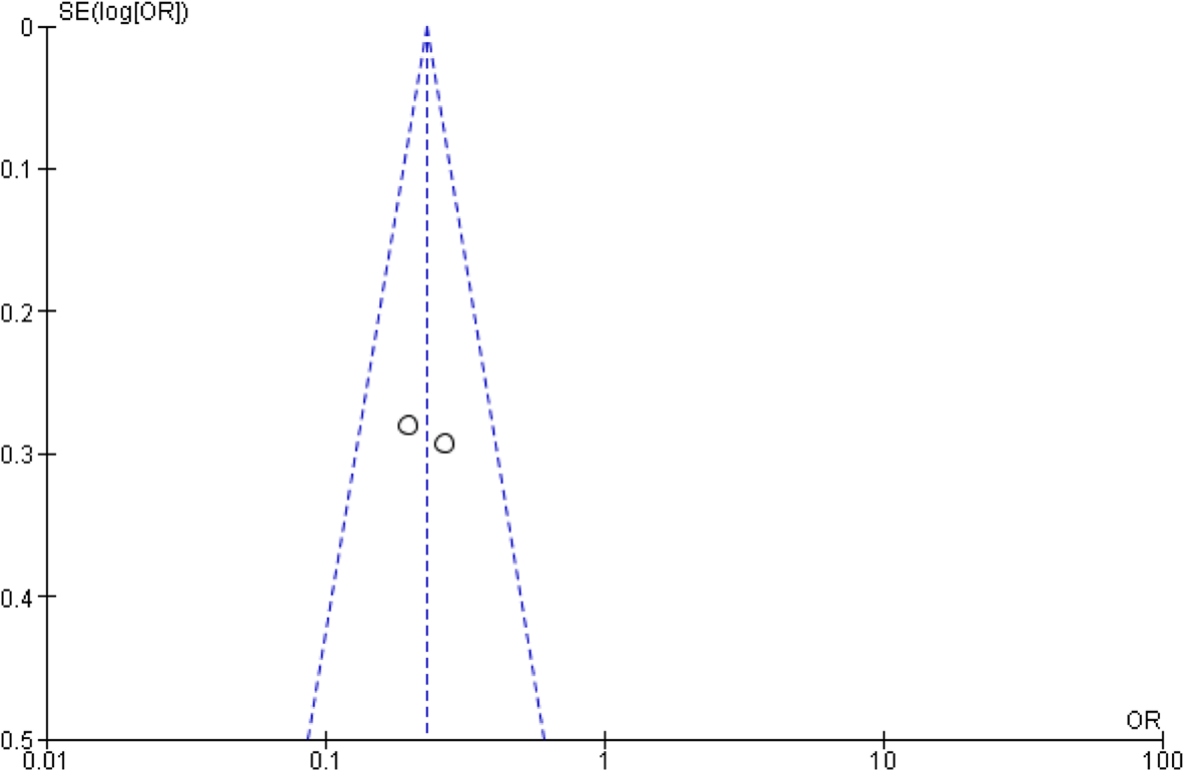
Funnel plot for meta-analysis of the association between parental acceptance to silver diamine fluoride application and type of primary teeth. Funnel plot shows homogeneous distribution of the two included studies

According to 3 studies [19–21], primary posterior teeth had a higher parental acceptance of SDF application compared to anterior teeth (Table 1); 2 of these studies [19, 20] were included in the meta-analysis. A forest plot for the meta-analysis found a significantly lower parental acceptance of SDF application on children’s primary anterior teeth compared to posterior teeth (*P* < 0.0001, OR: 0.25 and 95% CI: 0.15–0.42) (Fig. 2). There was low heterogeneity between the two studies (I^2^ = 37%), no statistical significance (*P* = 0.21) and funnel plot showed homogeneous distribution of the two included studies (Fig. 3).

However, Alshammari et al. [21] reported that all parents refused SDF treatment for their children’s teeth regardless of the location, except for 3.2% who were neutral (Table 1). Belotti et al. [23] reported that 64.3% of parents noticed aesthetic differences only on primary posterior teeth, and all agreed that SDF treatment did not interfere with aesthetics. However, it was not possible to include the two mentioned studies in the meta-analysis since some of the subgroups contained zero values or did not compare between anterior and posterior teeth [21, 27].

### Patient cooperation (cooperative vs. uncooperative)

**Fig. 4:**
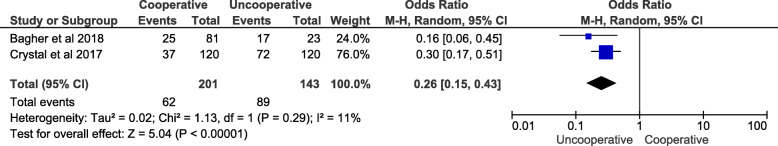
Forest plot for meta-analysis of the association between parental acceptance to silver diamine fluoride application on primary anterior teeth and child cooperation

The relationship between child cooperation and parental acceptance of SDF was compared according to tooth location (anterior or posterior) in 2 studies [19, 20], and both were entered into the meta-analysis. The results demonstrated a significant relationship between child cooperation and parental acceptance of SDF for anterior teeth (*P* < 0.0001, OR: 0.26 and 95% CI: 0.15–0.43; Fig. 4). However, although Bagher et al. [20] reported a significant relationship between child cooperation and parental acceptance of SDF on posterior teeth (*P* < 0.05), the meta-analysis found no significant relationship overall (*P* = 0.42, OR: 0.12 and 95% CI: 0.00–22.13; supplementary Fig. 1). There was low heterogeneity between the two studies (I^2^ = 11%) with no statistical significance (*P* = 0.29) on measuring the relationship of child cooperation with anterior teeth. However, this was not the case with posterior teeth, which displayed a statistically significant high heterogeneity (*P* < 0.0001).

### Follow-up and concentration

One randomized controlled study reported that parental acceptance rate for SDF use increased with time in the follow-up visits [25], from 1.4% at baseline to 4.5% at 30 months follow-up. In addition, Daungthip et al. [25] was the only study that compared parental acceptance for 2 different SDF concentrations and found no statistically significant difference in parental acceptance between 12 and 38% SDF concentration (*P* > 0.05; Table 1).

### Taste, and SDF application

Clements et al. [12] reported that parental acceptance fell into either the acceptable or somewhat-acceptable range for taste (63.3 and 23.3%, respectively), discoloration (53.3, and 33.3%, respectively), and application process (63.3 and 26.7%, respectively). Moreover, 70.0% of the parents reported that SDF application was pain-free and 33.3% reported that it was somewhat pain-free for their children (Table 1).

### Instruction lecture before application of SDF

Triches et al. [27] divided the study sample set into 2 groups before answering the questionnaire; 1 group received instructions regarding the indication and usage of SDF, while the other group did not receive any prior instruction and answered the questionnaire directly after viewing the image. The group that received instructions had a lower resistance (12%) to SDF treatment compared to the group that did not receive instructions (28%), if the percentages of somewhat-unacceptable and unacceptable groups were combined together (Table 1).

### Strength of reporting, quality assessment and risk of bias

The only article sections assessed for qualification or discussion of the treatment outcomes of SDF were those involving parental preference. The strength of reporting by the STROBE checklist demonstrated that two studies were of high-strength [19, 20], while the other three studies were of moderate-strength [21, 22, 27]. The key items affecting the STROBE checklist were the absence of a description of any efforts to address potential sources of bias [20–22, 27] and the study size and statistical method [21, 22, 27] (see end of manuscript, Supplemental Table S2). The CONSORT checklist showed that Two studies were of High strength [24, 26] and three studies were of moderate-strength [12, 23, 25]. Crucial items affecting the CONSORT checklist were missing identification on the generation of the random allocation sequence and a lack of information on how the participants were enrolled and assigned to interventions [12, 23–25] (see end of manuscript, Supplemental Table S3). As for risk of bias, Duangthip et al. (2018) [25] showed low risk of bias in all items except in blinding the outcome “Detection bias”. See Table 2. In addition, Jadad scoring and NOQ quality assessment tools showed high range of quality scoring for Zhi et al. (2012) [24], Duangthip et al. (2018) [25] and Vollú et al. (2019) [26]. Other studies showed either moderate or low range of quality scoring (see supplementary Table S4 and S5).

**Table 2:** Risk of bias summary: review authors’ judgements for each included study according to each risk of bias item

Domain	Zhi et al. (2012)	Belotti et al. (2016)	Clements et al. (2017)	Duangthip et al. (2017)	Vollú et al.(2019)
**Selection bias** Random sequence generation	**–**	**+**	**+**	**–**	**–**
**Selection bias** Allocation concealment	**+**	**+**	**+**	**–**	**–**
**Reporting bias** Selective reporting	**+**	**+**	**+**	**–**	**–**
**Other bias** Other sources of bias	**+**	**+**	**+**	**–**	**–**
**Performance bias** Blinding (participants and personnel)	**+**	**?**	**+**	**–**	**+**
**Detection bias** Blinding (outcome assessment)	**+**	**?**	**+**	**?**	**+**
**Attrition bias** Incomplete outcome data	**+**	**?**	**–**	**–**	**–**

Quality items distributed to: high risk of bias”+”, low risk of bias “-”, and unclear “?”

### Category of evidence and strength of recommendation of the included articles

Three of the result topics including tooth location, patient’s cooperation, and instruction lecture before SDF application, fall under category III of evidence according to the guidelines devised by Shekelle et al. [28] since the included studies reported evidence obtained from non-experimental descriptive studies, such as comparative studies, correlation studies, cohort studies, and case-controlled studies. In addition, all 3 results were considered class C, based on category III evidence, according to the guidelines. Two of the results in the “follow-up and concentration” group fell under category Ib of evidence since they reported evidence from at least 1 RCT. In addition, these 2 results were considered class A based on category I evidence. Moreover, results of taste, appearance, application, and amount of discomfort were considered category IIa since they included evidence from at least 1 controlled study without randomization. They were considered class B directly based on category II evidence, according to the guidelines used by Shekelle et al. [28] (see Table 3 at the end of the manuscript).

**Table 3:** Evidence of SDF parental preference category and recommendation strength

Topic	Recommendation	Evidence Category	Recommendation Strength
Tooth location (anterior vs. posterior)	Parental preference of SDF in posterior teeth is higher than anterior teeth.	III***	C^^^
Patient’s cooperation (cooperative vs. uncooperative)	Parental preference of SDF use in anterior teeth for non-cooperative children is higher than in cooperative children.	III***	C^^^
Follow-up	Parental acceptance rate for SDF use increased with time.	Ib*	A^
Concentration	No difference in parental acceptance between 12 and 38% SDF concentrations.	Ib*	A^
Taste, appearance, application, and amount of discomfort	Most parents found the taste, appearance, application process, and amount of discomfort to their children acceptable.	IIa**	B^^
Instruction lecture before application of SDF^^^^	The group that received an instruction lecture had a lower resistance to SDF treatment compared to the non-instruction group.	III***	C^^^

Notes: This table is according to the recommendation system of Shekelle et al .[27]

* Category Ib is evidence from at least one randomized controlled trial

**Category IIa is evidence from at least one controlled study without randomization

***Category III is evidence from non-experimental descriptive studies, such as comparative studies, correlation studies, and case-control studies

^Class A is directly based on category I evidence

^^Class B is directly based on category II evidence

^^^Class C is directly based on category III

^^^^Only 1 study was available for the recommendation

## Discussion

Our review included ten reports on parental acceptance of SDF treatment for primary teeth. We found that the key factors affecting parental acceptance were tooth location and child cooperation.

Parental preference and acceptance of the type of tooth restoration for their children’s caries affect the everyday working life of a dentist. SDF has been reintroduced to the dental market and retains many advantages including carious prevention, but it has the drawback of tooth discoloration [7, 8, 11]. According to Zhi et al. [24], the easy application procedure of SDF relative to its counterparts, such as drilling and filling (which are distasteful to some patients), is an advantage [29]. Parents had statistically significant higher acceptance of SDF when used on posterior teeth. This phenomenon is similar to other types of unaesthetic restorations such as stainless-steel crowns [30], since typically the patient prefers a more aesthetic restoration when it is more visible [31]. The practitioner may take this into account by considering SDF as a treatment option for posterior teeth rather than anterior teeth. Also, after consecutive follow-ups, parental satisfaction with the treatment increased [25]. This phenomenon was observed with other types of aesthetic procedures such as facial plastic surgery [32] and may be due in part to the desensitization and adaptation of the brain to visual stimuli over time [33]. Crystal et al. [19] and Bagher et al. [20] reported that parents might be more accepting of SDF if their child was uncooperative, especially on anterior teeth. This finding is important because it shows that parents tend to avoid more severe behavior management strategies, such as general anesthesia or passive restraint, which are viewed as unfavorable [34]. Clemens et al. [12] stated that child behavior during SDF application was not correlated with subjective parent feelings about the discoloration of teeth. However, we did not include that study in the meta-analysis since no data were available for measurement. Thus, the practitioner may present SDF as an option to the parent before more invasive methods are considered. Offering SDF as an option before considering general anesthesia is especially relevant in preventing the associated side effects [35]. Two articles from Brazil [23, 27] were included in our study. The convenience and cost-effectiveness of SDF makes it a very favorable treatment for children of lower socioeconomic status. This may partially explain the high acceptance rate of SDF in Brazil since parents may not have other affordable options for treating and relieving pain in their children. Moreover, Triches et al. [27] reported that parents instructed on the indications and usage of SDF were more accepting of SDF than those who were not aware of the implications and advantages of SDF. This confirms the importance of parental education since many parents may associate a dark appearance of teeth with caries or poor oral hygiene. According to Clements et al. [12], most parents found the taste, appearance, application process, and amount of discomfort to their child acceptable. The taste acceptability is especially interesting since children typically have a low acceptance of bitter-tasting medicaments [36], and SDF has a bitter metallic taste [10]. The acceptance may be due to the relatively short time of the treatment application, but further improvements in taste by adding flavoring agents may be recommended.

However, the limitation and the high risk of bias in most of included studies recommend future RCTs studies, that assess parental acceptance to SDF in primary and permanent teeth, especially for medically compromised patients, and in low socioeconomic communities.

## Conclusion

Parental acceptance to SDF treatment as statistically significantly related to the location of teeth and child’s cooperation; it was significantly higher in posterior teeth compared to anterior teeth and in uncooperative children compared to cooperative children. Parental pre-operative instruction also significantly improved parental acceptance to SDF treatment.

This review will have a positive effect on the implementation of treatments for children’s dental caries in future since parental acceptance is a major factor in choosing SDF.

## Supplementary information

**Additional file 1: Supplementary Table S1.** Definitions of “parental acceptance” and scales reported by each included study. **Supplementary Table S2.** Evaluation of included case-control and cross-sectional studies content according to STROBE (2007) checklist. **Supplementary Table S3.** Evaluation of the included clinical trials content according to CONSORT (2010) Check list. Supplementary **Table S4.** Quality assessment according to Oxford Quality Scoring System for clinical trial studies. **Supplementary Table S5.** Quality assessment according to Newcastle-Ottawa Quality Assessment Form for Case-Control Studies.

**Additional file 2: Supplementary Figure 1.** Forest plot for meta-analysis of the association between parental acceptance to silver diamine fluoride application on primary posterior teeth and child cooperation.

## Data Availability

All data generated or analysed during this study are included in this published article.

## Abbreviations

SDF: Silver diamine flouride
STROBE: Strengthening the reporting of observational studies in epidemiology
CONSORT: CONsilidated standards of reporting trials
RCT: Randomized controled trials
