# Supplementary material for: Parental acceptance of silver Diamine fluoride application on primary dentition: a systematic review and meta-analysis
Source: BMC Oral Health. 2020 Aug 20;20:227. doi: 10.1186/s12903-020-01195-3 (PMC7439720; doi:10.1186/s12903-020-01195-3)
Supplement: Supplementary file 1 — Additional file 1: Supplementary Table S1. Definitions of “parental acceptance” and scales reported by each included study. Supplementary Table S2. Evaluation of included case-control and cross-sectional studies content according to STROBE (2007) checklist. Supplementary Table S3. Evaluation of the included clinical trials content according to CONSORT (2010) Check list. Supplementary Table S4. Quality assessment according to Oxford Quality Scoring System for clinical trial studies. Supplementary Table S5. Quality assessment according to Newcastle-Ottawa Quality Assessment Form for Case-Control Studies. [file 12903_2020_1195_MOESM1_ESM.docx]

**Supplementary table 1 “****Table S1”: Definitions of “parental acceptance” and scales reported by each included study**

| **Study** | **Definition of parental acceptance and questions** | **Scales** |
| --- | --- | --- |
| Kumar et al (2019) | -Parental perception of the black stain left by the SDF  -Level of parental comfort before their children received the SDF treatment | 5-point Likert scale ranging from 1=extremely concerned to 5=not concerned  5-point Likert scale ranging from 1 = very comfortable to 5 = very uncomfortable.  Perceived health- |
| Vollu et al (2019) | -Parents annoyed by the appearance of their children's teeth | Yes/ No |
| Alshammari et al., (2019) | -Before and after photos with questionnaire on parental SDF aesthetic acceptance | 5-point Likert scale ranging from 1=strongly accept to 5=strongly refuse. |
| Duangthip et al., (2017) | -Questionnaire regarding parental satisfaction with child’s dental appearance at baseline, 18, 30 months follow-up | 5-point Likert scale ranging from 1= Very satisfied to 5=very not satisfied |
| Bagher et al., (2018) | -Before and after photos with questionnaire on parental preference | 5-point Likert scale ranging from 1=strongly acceptable to 5=strongly unacceptable. |
| Crystal et al., (2017) | -Before and after treatment sets of photos then questionnaire to evaluate parents’ acceptance of the aesthetics  (primary teeth photographs | 4-point Likert scale ranging from 1=acceptable to 4=unacceptable. |
| Clements et al., (2017) | - Questionnaire on Parental satisfaction toward: SDF treatment, discoloration, easy application process, pain and taste | 5-point Likert scale ranging from 1=strongly agree to 5=strongly disagree. |
| Belotti et al., (2016) | parents evaluate the aesthetics acceptability | 3-point Likert scale ranging from 1=acceptable to 3=unacceptable |
| Zhi et al (2012) | Questionnaire on parent aesthetic satisfaction | Yes/ No |
| Triches et al (2009) | -Questionnaire on parent aesthetic satisfaction “are you satisfied?” | 5-point Likert scale ranging from 1=strongly agree to 5=strongly disagree. |

**Supplementary table 2** “**Table S2”: Evaluation of included case-control and cross-sectional studies content according to STROBE (2007) checklist**

| Paper | Crystal et al. (2017) [22] | Bagher et al. (2018) [25] | Alshammari et al. (2019) [30] | Triches et al. (2009) [31] | Kumar et al. (2019) [34] |
| --- | --- | --- | --- | --- | --- |
| Method |  |  |  |  |  |
| Study design (1) | 0 | 1 | 1 | 0 | 1 |
| Setting (3) | 1 | 3 | 0.5 | 3 | 2.5 |
| Participants (1) | 1 | 0.5 | 0 | 0.5 | 1 |
| Variables (1) | 0.5 | 1 | 0.5 | 0.5 | 0.5 |
| Data sources/ measurement (1) | 1 | 1 | 1 | 1 | 0.5 |
| Bias (1) | 1 | 0 | 0 | 0 | 0 |
| Study size (1) | 1 | 1 | 0 | 0 | 1 |
| Quantitative variables (1) | 1 | 1 | 1 | 1 | 1 |
| Statistical methods (4) | 4 | 4 | 1 | 1 | 2 |
| Results |  |  |  |  |  |
| Participants (2) | 2 | 1 | 2 | 2 | 2 |
| Descriptive data (2) | 2 | 1 | 2 | 1 | 2 |
| Outcome data (1) | 0 | 1 | 1 | 1 | 1 |
| Main results (2) | 2 | 1.5 | 1 | 0 | 1 |
| Other analyses (1) | 1 | 1 | 0 | 0 | 0 |
| Score | 17.5 / 22  High strength | 18/ 22  High strength | 11/22  Moderate strength | 11/ 22  Moderate strength | 15.5/ 22  Moderate strength |

1–7 (poor strength), 8–15 (moderate strength) and 16–22 (high strength)

**Supplementary table 3** “**Table S3”: Evaluation of the included clinical trials content according to CONSORT (2010) Check list**

| Paper | Clements et al. (2017) [12] | Zhi et al. (2012) [23] | Duangthip et al. (2018) [24] | Belotti et al. (2016)  [32] | Vollú et al. (2019) [33] |
| --- | --- | --- | --- | --- | --- |
| Method |  |  |  |  |  |
| Trial design (2) | 2 | 2 | 1 | 2 | 2 |
| Participants (2) | 1.5 | 2 | 2 | 1.5 | 2 |
| Interventions (1) | 1 | 0.5 | 1 | 0.5 | 1 |
| Outcomes (2) | 1.5 | 0.5 | 1 | 1.5 | 2 |
| Sample size (2) | 1 | 2 | 1 | 2 | 2 |
| Randomization (4) | 0 | 3 | 2 | 0 | 4 |
| Blinding (2) | 0 | 2 | 2 | 1 | 0.5 |
| Statistical methods (2) | 2 | 1 | 2 | 0 | 1 |
| Results |  |  |  |  |  |
| Participants (2) | 1 | 2 | 2 | 1 | 2 |
| Recruitment (1) | 1 | 1 | 0 | 1 | 0.5 |
| Why the trial ended or was stopped (1) | 1 | 1 | 0 | 1 | 0 |
| baseline data (1) | 1 | 1 | 0 | 1 | 1 |
| Number analyzed (1) | 1 | 1 | 1 | 1 | 1 |
| Outcomes (2) | 0.5 | 0 | 1 | 0 | 2 |
| Ancillary analysis (1) | 0 | 0 | 1 | 0 | 0 |
| harm (1) | 0 | 0 | 1 | 0 | 1 |
| Score out of 27 | 14.5/ 27  Moderate strength | 19/ 27  High strength | 18/27  Moderate strength | 13.5 / 27  Moderate strength | 22/27  High strength |

1–9 (poor strength), 10–18 (moderate strength) and 19–27 (high strength)

**Supplementary table 4** “**Table S4”Quality assessment according to** **Oxford Quality Scoring System for clinical trial studies**

| Paper | Clements et al. (2017) [12] | Zhi et al. (2012) [23] | Duangthip et al. (2018) [24] | Belotti et al. (2016)  [32] | Vollú et al. (2019) [33] |
| --- | --- | --- | --- | --- | --- |
| Method |  |  |  |  |  |
| 1. Was the study described as random? | 0 (no) | 1 (yes) | 1 (yes) | 0 (no) | 1 (yes) |
| 2. Was the randomization scheme described and appropriate? | -1 (no) | 1 (yes) | 1 (yes) | -1 (no) | 1 (yes) |
| 3. Was the study described as double-blind? | IN | IN | IN | IN | IN |
| 4. Was the method of double blinding appropriate? | IN | IN | IN | IN | IN |
| 5. Was there a description of dropouts and withdrawals? | 1 (yes) | 1 (yes) | 1 (yes) | 0 (no) | 1 (yes) |
| Score out of 3 | -1  low range of quality score | 3  high range of quality score | 3  high range of quality score | -1  low range of quality score | 3  high range of quality score |

≤2 (Low range of quality score), 3 (High range of quality score), IN: inapplicable

**Supplementary table** “**Table S5”: Quality assessment according to Newcastle-Ottawa Quality Assessment Form for Case-Control Studies**

| Paper | Triches et al. (2009) [31] |
| --- | --- |
| Selection |  |
| Adequate case definition (1) | 1 |
| Representativeness of the cases (1) | 0 |
| Selection of controls (1) | 0 |
| Definition of controls (1) | 0 |
| Comparability |  |
| Comparability of cases and controls on the basis of the design or analysis controlled for confounders (2) | 0 |
| Exposure |  |
| Ascertainment of exposure (1) | 0 |
| Same method of ascertainment for cases and controls (1) | 1 |
| Non-response rate (1) | 1 |
| Score out of 9 stars | 3 |

3-6 (fair range of quality score), <3 (poor quality)
