# Supplementary material for: Parental acceptance of silver Diamine fluoride application on primary dentition: a systematic review and meta-analysis
Source: BMC Oral Health. 2020 Aug 20;20:227. doi: 10.1186/s12903-020-01195-3 (PMC7439720; doi:10.1186/s12903-020-01195-3)
Supplement: Supplementary file 2 — Additional file 2: Supplementary Figure 1. Forest plot for meta-analysis of the association between parental acceptance to silver diamine fluoride application on primary posterior teeth and child cooperation. [file 12903_2020_1195_MOESM2_ESM.docx]

Supplementary Figure 1: Forest plot for meta-analysis of the association between parental acceptance to silver diamine fluoride application on primary posterior teeth and child cooperation.
